# Predictors of Treatment Response to Tesamorelin, a Growth Hormone-Releasing Factor Analog, in HIV-Infected Patients with Excess Abdominal Fat

**DOI:** 10.1371/journal.pone.0140358

**Published:** 2015-10-12

**Authors:** Alexandra Mangili, Julian Falutz, Jean-Claude Mamputu, Miganush Stepanians, Brooke Hayward

**Affiliations:** 1 EMD Serono, Inc., Rockland, Massachusetts, United States of America; 2 HIV Metabolic Unit, McGill University Health Centre, Montreal, Quebec, Canada; 3 Theratechnologies Inc., Montreal, Quebec, Canada; 4 PROMETRIKA, Cambridge, Massachusetts, United States of America; College of Medicine, National Taiwan University, TAIWAN

## Abstract

**Background:**

Tesamorelin, a synthetic analog of human growth hormone-releasing factor, decreases visceral adipose tissue (VAT) in human immunodeficiency virus (HIV)-infected patients with lipodystrophy.

**Objectives:**

1) To evaluate the utility of patient characteristics and validated disease-risk scores, namely indicator variables for the metabolic syndrome defined by the International Diabetes Federation (MetS-IDF) or the National Cholesterol Education Program (MetS-NCEP) and the Framingham Risk Score (FRS), as predictors of VAT reduction during tesamorelin therapy at 3 and 6 months, and 2) To explore the characteristics of patients who reached a threshold of VAT <140 cm^2^, a level associated with lower risk of adverse health outcomes, after 6 months of treatment with tesamorelin.

**Methods:**

Data were analyzed from two Phase 3 studies in which HIV-infected patients with excess abdominal fat were randomized in a 2:1 ratio to receive tesamorelin 2 mg (n = 543) or placebo (n = 263) subcutaneously daily for 6 months, using ANOVA and ANCOVA models.

**Results:**

Metabolic syndrome (MetS-IDF or MetS-NCEP) and FRS were significantly associated with VAT at baseline. Presence of metabolic syndrome ([MetS-NCEP), triglyceride levels >1.7 mmol/L, and white race had a significant impact on likelihood of response to tesamorelin after 6 months of therapy (interaction *p*-values 0.054, 0.063, and 0.025, respectively). No predictive factors were identified at 3 months. The odds of a VAT reduction to <140 cm^2^ for subjects treated with tesamorelin was 3.9 times greater than that of subjects randomized to placebo after controlling for study, gender, baseline body mass index (BMI) and baseline VAT (95% confidence interval [CI] 2.03; 7.44).

**Conclusions:**

Individuals with baseline MetS-NCEP, elevated triglyceride levels, or white race were most likely to experience reductions in VAT after 6 months of tesamorelin treatment. The odds of response of VAT <140 cm^2^ was 3.9 times greater for tesamorelin-treated patients than that of patients receiving placebo.

## Introduction

The long-term use of antiretroviral therapy (ART) in patients with human immunodeficiency virus (HIV) is associated with the variable development of increased visceral adiposity (lipohypertrophy), loss of subcutaneous fat (lipoatrophy), dyslipidemia, and insulin resistance. These changes in body shape and metabolism are sometimes termed ‘HIV-associated lipodystrophy syndrome’ [1–4]. These changes in body shape may negatively impact adherence to ART, as well as patients’ perspective of their body image and their quality of life [5–10]. Of potentially greater concern is an increased risk of type 2 diabetes (T2DM) and cardiovascular diseases (CVD) in patients with this syndrome [11–15]. Increased visceral adipose tissue (VAT) is commonly seen in patients with increased waist circumference (WC). Both of these factors are associated with metabolic abnormalities and are independent predictors of T2DM, CVD and mortality in HIV-negative patients [16–23]. In HIV-infected patients, increased VAT is associated with an adverse lipid profile, elevated Framingham Risk Score (FRS), and increased mortality [24–27]. Furthermore, VAT is positively correlated with greater baseline and greater rate of progression of coronary artery calcium, a surrogate marker of atherosclerosis [28–29].

Tesamorelin (Theratechnologies, Inc., Montreal, Quebec, Canada) is a synthetic analog of human growth hormone-releasing factor (also known as growth hormone-releasing hormone, GHRH), which is indicated for the treatment of excess abdominal fat in HIV-infected patients with lipodystrophy. The basis of the current report was a pooled analysis of the two pivotal, randomized Phase 3 trials in 806 ART-treated patients with HIV and excess abdominal fat, who were randomized 2:1 to receive tesamorelin 2 mg (*n* = 543) or placebo (*n* = 263) subcutaneously (sc) daily. Efficacy and safety outcomes of these studies have been described previously [30–32].

In the current analysis, we investigated tools to identify patients who are likely to respond to therapy with tesamorelin, using disease-risk scores previously applied to HIV-infected cohorts, and characterized patients who reached a threshold of VAT <140 cm^2^ after 6 months of treatment.

## Methods

### Study Design

These exploratory analyses aimed to construct a statistical model to identify patients likely to respond to tesamorelin after 3 or 6 months of therapy in the clinical setting. The model used easily measurable characteristics and standard disease-risk scores previously applied to HIV-infected cohorts, including indicator variables for metabolic syndrome (MetS), defined by the International Diabetes Federation (MetS-IDF) and defined by the National Cholesterol Education Program (MetS-NCEP), as well as the Framingham Risk Score (FRS) [33–35]. The primary analysis assessed the association between absolute change in VAT from baseline to 3 months and from baseline to 6 months, and baseline covariates. Selected baseline covariates for the analysis were: FRS (continuous and dichotomous: FRS ≥10% vs. FRS <10%), MetS-NCEP (present vs. absent), MetS-IDF (present vs. absent), age, gender, race, body mass index (BMI), WC, waist-to-hip ratio, weight, blood pressure (BP), cholesterol, triglycerides, duration of HIV infection, duration of ART, protease inhibitor (PI)-based highly active antiretroviral therapy (HAART), non-nucleoside reverse transcriptase inhibitor-based HAART, and other medication use. The secondary analysis explored the characteristics of subjects who reached a threshold of VAT <140 cm^2^ after treatment for 6 months, as this level has been associated with lower risk for adverse outcomes [23, 36, 37].

### Subjects

The patient population for both studies pooled for these analyses comprised HIV-infected individuals receiving stable ART aged 18–65 years who had abdominal fat accumulation (defined as patients with a WC of ≥ 95 cm for men and ≥ 94 cm for women plus an elevated waist-to-hip ratio of ≥ 0.94 for men and ≥ 0.88 for women) [38]. Women with a normal mammogram within 6 months of study and not pregnant were included. Subjects were excluded with (1) BMI < 20 kg/m^2^; (2) HIV-related disease/ infection within 3 months of study; (3) history of malignancy or active neoplasm; (4) prostate specific antigen (PSA) > 5 μg/L; (5) history of pituitary tumor/- surgery or head irradiation; (6) untreated hypothyroidism; (7) prior use of insulin, oral hypoglycemic, or insulin sensitizing agent witin 6 months of study; (8) alanine aminotransferase or aspartate aminotransferase > 3 x normal; (9) creatinine > 133 μmol/L; (10) hemoglobin > 20 g/L below normal; (11) fasting blood glucose ≥ 8.33 mmol/L, known history of Type I diabetes mellitus or Type II diabetes mellitus requiring medication; (12) fasting triglycerides > 11.3 mmol/L or change in lipid-lowering regimen within 3 months before study; (13) untreated hypertension; (14) change in testosterone regimen and/or supraphysiological dose of testosterone; (15) estrogen therapy; (16) anoretics/anorexigenics or anti-obesity agents within 3 months of study; growth hormone (GH), GH secretagogues, GHRH products, insulin-like growth factor-1 (IGF-1), or insulin-like growth factor-binding protein-3 (IGFBP-3) within 6 months of study; (18) drug or alcohol dependence or use of methadone within 6 months of study entry; and (19) participation in a clinical trial with any investigational drug/device within 30 days of screening.

### Assessment of Visceral Adipose Tissue

Visceral adipose tissue (VAT) was assessed by computed tomography (CT) scan from a single 5-mm slice obtained at the level of L4-L5 intervertebral disc. Images were analyzed in a blinded fashion at a Central Imaging Reading Centre (Perceptive Informatics, Waltham, MA, USA).

### Statistical Analysis

Exploratory analyses were conducted in the intent-to-treat (ITT) populations of two previously reported Phase 3 studies (NCT00123253 and NCT00435136) [39–40]. Both of the Phase 3 studies included a randomized placebo-controlled 6-month primary phase followed by a safety extension. The ITT analysis set was defined as all randomized subjects who received at least one dose of study treatment. The analyses reported here were performed for the integrated ITT populations of both trials. All models included a factor for study to adjust for any differences between trials.

The following methodologies for statistical modeling were pre-specified in a post-hoc statistical analysis plan. ANOVA and ANCOVA models were built for baseline VAT with effects for the dichotomized baseline FRS (<10% vs. ≥10%), study, baseline WC, and the FRS-by-WC interaction effect and their interaction. To test the homogeneity of results between the two trials, the interaction effects were tested at α = 0.1 and omitted from the model if not significant. The ANOVA and ANCOVA modeling were repeated using the composite-risk variables MetS-NCEP and MetS-IDF, in place of FRS, and with BMI replacing WC.

A second ANOVA model was built for absolute change in VAT from baseline to 3 months and 6 months with effects for study, treatment, baseline FRS (<10% vs. ≥10%), and the interaction of baseline FRS and treatment. If the interaction was significant at α = 0.1, subset analyses were conducted by FRS <10% and FRS ≥10%. The ANOVA analyses for absolute change in VAT from baseline to 3 months and 6 months were subsequently repeated, using the composite-risk variables MetS-IDF and MetS-NCEP and other key covariates, instead of FRS.

### Ethics Statement

The protocols of the two Phase 3 studies were approved by the institutional review board (IRB) used by the participating sites. These IRBs included: Biomedical Research Ethics Board Montreal General Hospital, Partners Human Research Committee IRB, IRB Services, St. Luke’s-Rooselvet Institute for Health, IRB AIDS Research Consortium of Atlanta, Western IRB, Human Subjects Protection Committee Medical IRB, IRB Rush University Medical Center, AIDS Research Alliance IRB, Kaiser Permanente Northern California IRB, Integrated Scientific and Ethical Review Board, Copernicus Group IRB, UBC Providence Health Care Research Ethics Board, IUPUI and Clarian IRB, New England IRB, Human Subjects Research Committee, Tufts New England Medical Center IRB, Committee for the Protection of Human Subjects, Human Research Protection Program University of California, Dallas VA Medical Center Human Studies Subcommittee, IRB University of Maryland, New York University School of Medicine Institutional Board of Research Associates, Conjoint Health Research Ethics Board University of Calgary Health Region, University of Cincinnati IRB, Research Ethics Board Sunnybrook Health Sciences Centre, Research Ethics Board, Fountain Valley Regional Hospital & Medical Center, Human Research Ethics Committee University of Sherbrooke, Hamilton Health Sciences, Clinical Research Ethics Committee CHUL, University Health Network Research Ethics Board, Florida Department of Health IRB, Office for the Protection of Human Subjects Medical IRB, Office of Human Research Ethics UNC Biomedical IRB, Committee on Human Research, The University of Texas Southwestern Medical Center IRB, IRB University Hospital Case Medical Center, UCSF Committee on Human Research, Tufts medical Center IRB, Ethics Committee CHU Sart-Tilman, CPP Ouest, Ethic Committee CEIC, and London MREC. All participants provided institutionally approved, written informed consent.

## Results

### Subject Baseline Characteristics

In total, 806 ART-treated subjects (ITT population) with HIV and anthropometric parameters consistent with excess intra-abdominal fat from two studies were included in the exploratory analyses to identify predictors of VAT response after 3 or 6 months of treatment with tesamorelin. Baseline characteristics were comparable for subjects who received tesamorelin and placebo in the two Phase 3 studies [39, 41]. Key baseline characteristics for the pooled analysis population are shown stratified by study in Table 1. Few statistical differences were seen between studies, confirming the similarity of design and allowing for pooling of individual data. No baseline differences between subjects receiving tesamorelin and placebo were revealed in the pooled analysis population after combining the studies. Baseline VAT was available for 802 individuals (*n* = 540 for tesamorelin and *n* = 262 for placebo). Subjects had a mean (standard deviation [SD]) VAT of 182.4 cm^2^ (83.5), consistent with markedly increased VAT. The mean (SD) FRS was 6.5% (4.3), suggestive of a low 1-year risk of CVD. Most subjects were male (85%) and Caucasian (76%). The prevalence of MetS-IDF was 73.4% and 62.8% had mean triglycerides >1.7 mmol/L. MetS-NCEP was present in 42.9% of individuals. Differences in the criteria for MetS-NCEP and MetS-IDF, including WC and number of other predictive components present, may account for the different frequencies of MetS estimated using these criteria [33, 34]. FRS and MetS (MetS-NCEP or MetS-IDF) were significantly associated with baseline VAT, even after controlling for WC (p<0.001 for all).

**Table 1:** Baseline Characteristics of each Study and the Pooled Analysis Population.

	Study 1 (N = 410)	Study 2 (N = 396)	Pooled Analysis Population (N = 806)
**Demographics**			
Age, years, mean (SD)	47.6 (7.4)	47.6 (7.6)	47.6 (7.5)
Males, *N* (%)	352 (85.9)	333 (84.1)	685 (85.0)
Race, *N* (%)			
Caucasian	308 (75.1)	305 (77.0)	613 (76.0)
Asian	2 (0.5)	3 (0.8)	5 (0.6)
African American	59 (14.4)	46 (11.6)	105 (13.0)
Hispanic	34 (8.3)	35 (8.8)	69 (8.6)
Other	7 (1.7)	7 (1.8)	14 (1.7)
**Disease and treatment history**			
Duration of HIV, years, mean (SD)^a^	13.3 (5.3)	14.0 (5.6)	13.6 (5.4)
Duration of ART, months, mean (SD)	53.7 (35.5)	52.8 (36.4)	53.3 (35.9)
PI-HAART, *N* (%)	229 (55.9)	216 (54.6)	445 (55.2)
**Body metrics**			
Weight, kg, mean (SD)	89.8 (13.9)	88.4 (14.3)	89.1 (14.1)
BMI, kg/m^2^, mean (SD)	29.2 (4.2)	28.8 (4.2)	29.0 (4.2)
BMI categories, kg/m^2^, *N* (%)			
Normal (18.5 to <25)	53 (12.9)	70 (17.7)	123 (15.3)
Overweight (25 to <30)	214 (52.2)	189 (47.7)	403 (50.0)
Obese (≥30)	143 (34.9)	137 (34.6)	280 (34.7)
WC, cm, mean (SD)	104.4 (9.5)	104.8 (9.0)	104.6 (9.3)
WC categories, *N* (%)			
High (>102 cm men, >88 cm women)	217 (52.9)	231 (58.3)	448 (55.6)
Low/moderate (≤102 cm men, ≤88 cm women)	193 (47.1)	165 (41.7)	358 (44.4)
Waist-to-hip ratio, mean (SD)	1.05 (0.06)	1.05 (0.07)	1.05 (0.07)
VAT, cm^2^, mean (SD)^a^	175.9 (76.9)	189.2 (89.5)	182.4 (83.5)
**Disease risk scores**			
FRS, mean (SD)^a^	6.1% (4.1%)	6.9% (4.4%)	6.5% (4.3%)
FRS categories, *N* (%)^a^			
FRS <10%	307 (74.9)	310 (78.3)	617 (76.6)
FRS ≥10%	43 (10.5)	78 (19.7)	121 (15.0)
Missing ^a^	60 (14.6)	8 (2.0)	68 (8.4)
MetS-NCEP, *N* (%)	176 (42.9)	170 (42.9)	346 (42.9)
MetS-IDF, *N* (%)	296 (72.2)	296 (74.8)	592 (73.4)
**Laboratory assessments**			
Triglycerides, mg/dL, mean (SD)	236.5 (153.9)	233.4 (230.5)	234.9 (195.5)
Triglycerides >1.7 mmol/L, *N* (%)	265 (64.6)	241 (60.9)	506 (62.8)
**Vital signs**			
Systolic BP, mmHg, mean (SD)^a^	124.6 (14.3)	122.2 (12.1)	123.4 (13.3)
Diastolic BP, mmHg, mean (SD)	79.0 (8.9)	78.3 (7.8)	78.7 (8.4)

Abbreviations: ART, antiretroviral therapy; BMI, body mass index; BP, blood pressure; FRS, Framingham Risk Score; HIV, human immunodeficiency virus; MetS-IDF, International Diabetes Foundation definition of metabolic syndrome; MetS-NCEP, National Cholesterol Education Program defined metabolic syndrome; PI–HAART, protease inhibitor-based highly active antiretroviral therapy; SD, standard deviation; VAT; visceral adipose tissue; WC, waist circumference.

^a^ Statistically significant difference between Study 1 and Study 2 (p<0.05 from Student’s t-test for continuous or Fisher’s exact test for categorical data). No differences between the treatment and placebo groups were seen within the pooled analysis population after combining the studies.

### Absolute Change in VAT

**Fig 1:**
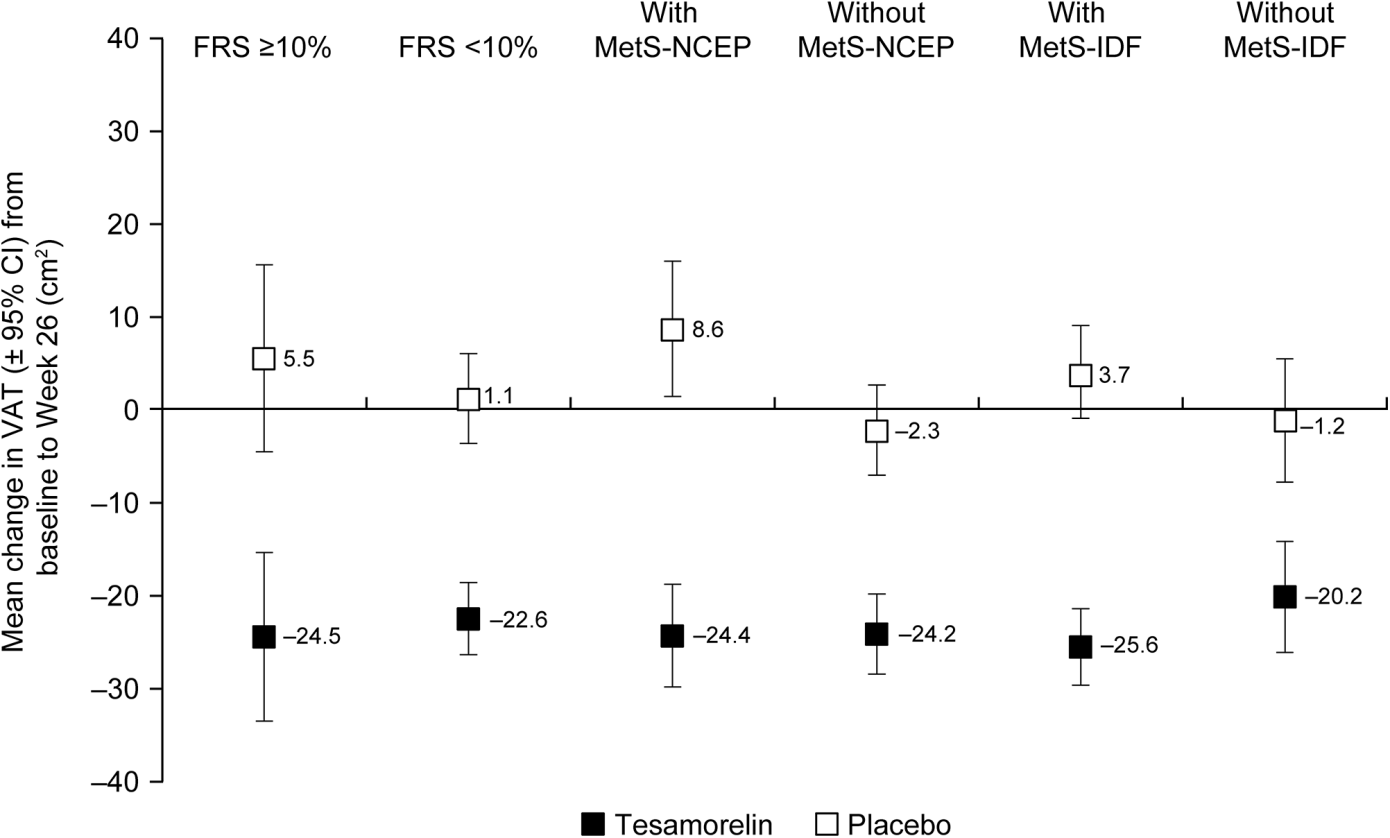
Mean change from baseline to 6 months in VAT by treatment and baseline disease-risk category. Baseline risk score (by FRS, MetS-NCEP or MetS-IDF) and the interaction between baseline risk score and treatment showed that the treatment effect of tesamorelin versus placebo was greater in patients with MetS-NCEP compared with those without MetS-NCEP (interaction p = 0.054). Abbreviation: FRS, Framingham Risk Score; MetS-IDF, International Diabetes Foundation definition of metabolic syndrome; MetS-NCEP, National Cholesterol Education Program-defined metabolic syndrome; VAT: visceral adipose tissue.

Mean changes in VAT from baseline to 6 months by treatment and risk category of FRS, MetS-NCEP and MetS-IDF are shown in Fig 1. The test of interaction showed that the treatment effect of tesamorelin versus placebo with respect to mean absolute change in VAT from baseline to 6 months was significantly greater (p = 0.054; α = 0.1) in patients with MetS-NCEP (–24.4 vs. 8.6 cm^2^) compared to those without MetS-NCEP (–24.2 vs. –2.3 cm^2^). For MetS-IDF, the interaction test did not reach statistical significance (p = 0.124; α = 0.1). However, the treatment effect of tesamorelin versus placebo was numerically greater for patients with MetS-IDF (–25.6 vs. 3.7 cm^2^) compared to those without MetS-IDF (–20.2 vs. –1.2 cm^2^). The treatment effect at 6 months was not statistically different for patients with baseline FRS ≥10% and those with baseline FRS <10% (p = 0.435; α = 0.1).

**Fig 2:**
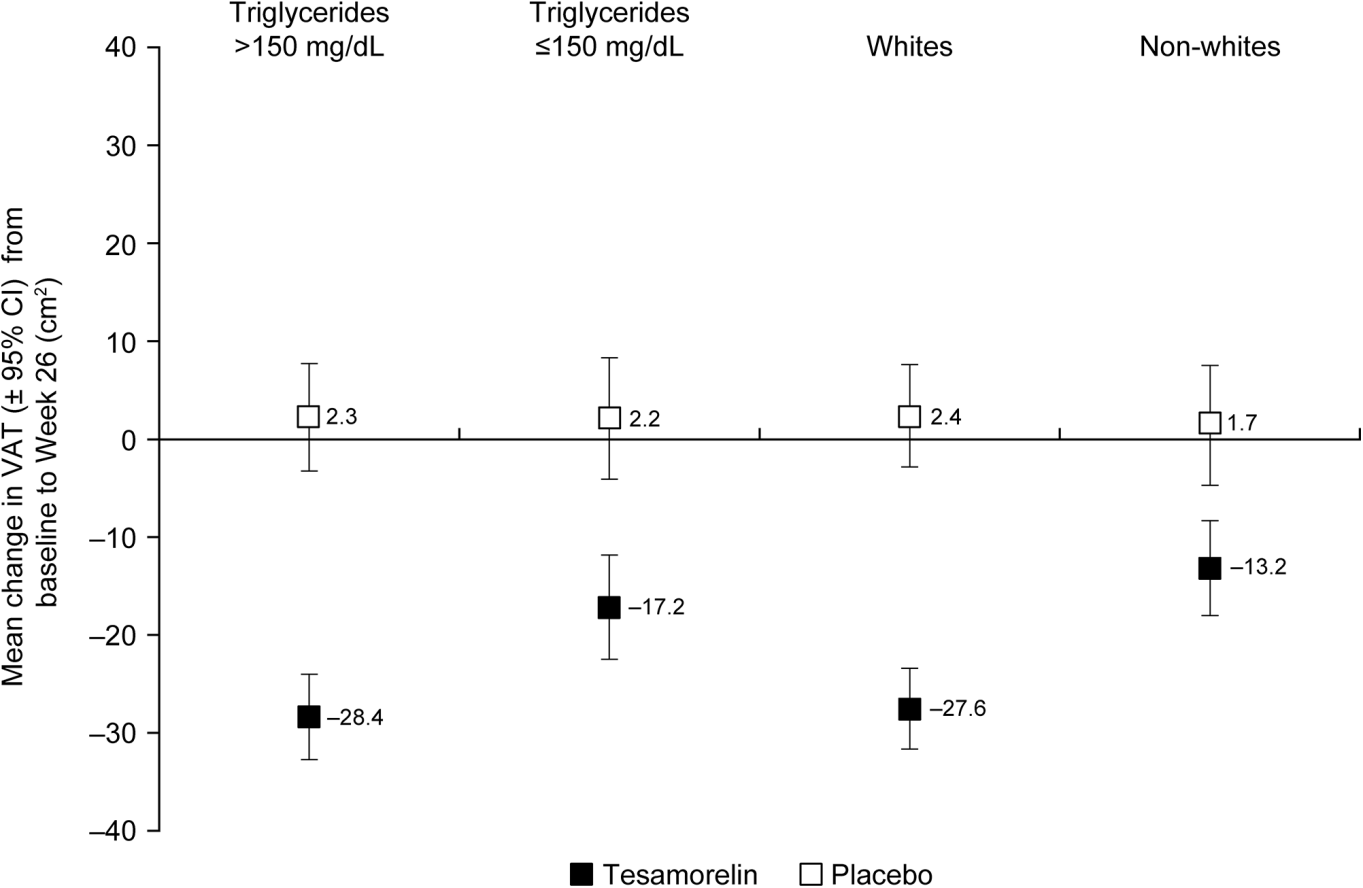
Mean change from baseline to 6 months by race and baseline triglyceride levels. The difference in mean absolute change in VAT from baseline to 6 months for tesamorelin versus placebo was greater for patients with triglycerides > 1.7 mmol/L than for those with triglycerides ≤ 1.7 mmol/L (interaction p = 0.063), and greater for white patients compared with non-white patients (interaction p = 0.025).

Baseline triglycerides (>1.7 mmol/L vs. ≤1.7 mmol/L) and race (white vs. non-white) were the only covariates that impacted treatment effect (interaction p = 0.063 and p = 0.025, respectively). As shown in Fig 2, the difference in mean absolute change in VAT from baseline to 6 months for tesamorelin versus placebo was larger for patients with triglycerides >1.7 mmol/L (–28.4 vs. 2.3 cm^2^) than for those with triglycerides ≤1.7 mmol/L (–17.2 vs. 2.2 cm^2^), and larger for white patients (–27.6 vs. 2.4 cm^2^) compared with non-white patients (–13.2 vs. 1.7 cm^2^). At 3 months, no significant interactions were observed between treatment and baseline covariates or disease-risk scores.

### Change in VAT to <140 cm^2^

The proportion of patients reaching a threshold of VAT <140 cm^2^ after 6 months of treatment is shown in Table 2. Different patterns of change in VAT were observed in the two treatment groups (p<0.0001). Of subjects with VAT ≥140 cm^2^ at baseline, 23% (*n* = 80 of 350) of those treated with tesamorelin for 6 months reduced their VAT to <140 cm^2^ compared with 9% (*n* = 14 of 163) of those receiving placebo. Furthermore, 44% of those with baseline VAT 140–200 cm^2^ and 7% of those with baseline VAT >200 cm^2^ had a follow-up VAT measurement of <140 cm^2^ following 6 months of treatment with tesamorelin, compared with 17% and 2% of those receiving placebo, respectively. Overall, of the 203 and 94 subjects receiving tesamorelin and placebo, respectively, who had baseline VAT ≥200 cm^2^, a greater proportion of those receiving tesamorelin achieved a reduction in VAT to <200 cm^2^ after 6 months (tesamorelin 34% [*n* = 70 of 203], placebo 16% [*n* = 15 of 94]). When stratified by BMI categories, 29% of those who were overweight and had a VAT ≥140 cm^2^ reduced their VAT to <140 cm^2^ after 6 months of treatment with tesamorelin compared with 24% and 15% of those who were normal weight or obese, respectively. For those receiving placebo, 11% of the overweight subjects who had a VAT ≥140 cm^2^ achieved a VAT reduction to <140 cm^2^ compared with 7% of subjects who were obese. The odds of a VAT reduction to <140 cm^2^ for subjects receiving tesamorelin was 3.9-times greater than that of subjects randomized to placebo after controlling for study, gender, baseline BMI and baseline VAT (95% confidence interval [CI] 2.03; 7.44). The odds of response of VAT <140 cm^2^ after 6 months in overweight subjects was 2.1 times greater than that of obese subjects after controlling for study, gender, baseline BMI, baseline VAT and treatment (95% CI 1.2; 3.8).

**Table 2:** Proportion of Patients Reaching a Threshold of VAT <140 cm^2^ by Treatment and BMI Category.

		**Baseline VAT (cm** ^**2**^ **)**					
**n (%)**		**Placebo (N = 263)**			**Tesamorelin (N = 543)**		
**Week 26 VAT (cm** ^**2**^ **)** ^a^		**<140**	**≥140**		**<140**	**≥140**	
**<140**		86 (86.0%)	14 (8.6%)		185 (95.9%)	80 (22.9%)	
		**<140**	**140 to <200**	**≥200**	**<140**	**140 to <200**	**≥200**
**<140**		86 (86.0%)	12 (17.4%)	2 (2.1%)	185 (95.9%)	65 (44.2%)	15 (7.4%)
**140 to <200**		12 (12.0%)	44 (63.8%)	13 (13.8%)	7 (3.6%)	70 (47.6%)	55 (27.1%)
**≥200**		2 (2.0%)	13 (18.8%)	79 (84.0%)	1 (0.5%)	12 (8.2%)	133 (65.5%)
**Total**		100	69	94	193	147	203
		**Baseline VAT (cm** ^**2**^ **)**					
**n/N (%)**		**Placebo (N = 263)**			**Tesamorelin (N = 543)**		
**Week 26 VAT (cm** ^**2**^ **)**	**BMI Category (kg/m** ^**2**^ **)**	**<140**	**≥140**		**<140**	**≥140**	
**<140**	**Normal (18.5 to <25)**	20/24 (83.3%)	1/19 (5.3%)		33/35 (94.3%)	11/45 (24.4%)	
	**Overweight (25 to <30)**	40/48 (83.3%)	9/85 (10.6%)		98/101 (97.0%)	49/169 (29.0%)	
	**Obese (≥30)**	26/28 (92.9%)	4/59 (6.8%)		54/57 (94.7%)	20/136 (14.7%)	

Abbreviations: BMI, body mass index; VAT; visceral adipose tissue.

^a^ Statistically significant difference in the pattern of change in VAT for patients on tesamorelin treatment vs. placebo (p<0.0001 from exact likelihood ratio test of association).

## Discussion

Tesamorelin has been shown to reduce VAT in HIV-infected patients with lipodystrophy. In this analysis, we identify some predictors of response to tesamorelin. Presence of MetS-NCEP, high triglycerides, and white race were associated with a greater likelihood of responding to 6 months of tesamorelin treatment. Although VAT was significantly correlated with both MetS-NCEP and MetS-IDF at baseline, even after controlling for WC, and both measures showed the same pattern of response to treatment, only MetS-NCEP reached the pre-specified level of significance. It appears that MetS-NCEP was a more sensitive indicator of response to tesamorelin treatment than MetS-IDF in our study population. Central obesity, as defined by WC, is a required component of the IDF definition, whereas WC is only one of five factors that define MetS-NCEP [42]. However, based on the inclusion criteria for these two Phase 3 trials, all participants with MetS-NCEP had high WC. Our data demonstrating that patients with MetS-IDF responded to tesamorelin treatment similarly to patients with MetS-NCEP suggest that presence of MetS-IDF can be used in the clinical setting to identify patients eligible to tesamorelin therapy. We also observed an apparent VAT increase in placebo-treated subjects with MetS or a high FRS over the 6-month period. This observation is indicative of a trend for increasing VAT in the untreated, at risk population. In a summary of eight recent studies in treatment-naïve, HIV-infected patients initiating highly active antiretroviral therapy (HAART), VAT increases in response to first-line treatment were variable, which led the author to conclude that VAT may still increase with current regimens [43]. Our finding demonstrating that presence of high triglyderides was a predictor of response to tesamorelin could be a reflexion of the documentated positive association between increased VAT and high triglycerides and WC in HIV-infected patients [44]. To our knowledge, there are no data in the scientific literature demonstrating genetic variability in the pharmacodynamic response to growth hormone-releasing factor or growth hormone. Nonetheless, data from a recent population pharmacokinetic analysis indicate that race does not affect tesamorelin pharmacokinetics in HIV-infected patients and healthy subjects [45]. No predictors of response were identified when data were examined at the earlier 3-month time point.

A level of VAT <140 cm^2^ has been associated with lower risk of adverse health outcomes [23, 36, 37]. In the current analysis, we identified a variable response to treatment depending on baseline VAT above a certain threshold, as well as by different BMI categories. The most robust response appears to be in subjects with VAT above 140 cm^2^ but below 200 cm^2^, as well as those in the overweight range for BMI measures. However, individuals with VAT >200 cm^2^ still lost a significant amount of VAT. Stanforth et al. [46] showed that a VAT-prediction model derived from the HERITAGE Family Study had decreasing accuracy with increasing abdominal visceral fat. It is possible that our tools to accurately identify VAT may be limited. For example, a CT scan at L4–L5 may not measure VAT with the same accuracy in the obese or highest VAT group who may have a more variable distribution of VAT, and hence the reduction with treatment may not be captured as precisely at the same vertebral level [47–49]. Our results demonstrating a decrease to level of VAT <140 cm^2^ in normal weight subjects (BMI 18.5 to <25 kg/m^2^) with VAT ≥140 cm^2^ at baseline may have important clinical implications as a significant proportion of HIV-infected patients may present with sarcopenic obesity [50], which could be due to a reduced GHRH secretion, resulting in decreased GH synthesis, and subsequently, lower hepatic production of IGF-1 [51]. Obesity, loss of muscle mass, and decreased IGF-1 levels are independently associated with disability and frailty in elderly subjects [52–54]. Data from the Study of Fat Redistribution and Metabolic Change in HIV infection (FRAM) showed that decreased limb muscle mass and increased VAT are associated with all-cause 5-year mortality in HIV-infected patients [27].

It is unclear if a reduction of VAT to below a critical threshold is necessary or if a clinical benefit can be achieved with any amount of VAT reduction, especially as this has not been studied in the HIV-infected population. Among overweight and obese individuals in the general population, the prevalence of hypertension, impaired fasting glucose levels, and MetS has been shown to increase linearly and significantly across increasing VAT quartiles [55–56]. The relationship between visceral adiposity and cardiovascular risk should be considered as a continuum [57–58], with an increased risk of metabolic abnormalities and cardiovascular endpoints generally occurring with VAT levels above the range of 130–150 cm^2^ [36, 37, 59]. In patients with HIV, increased VAT has been associated with atherogenic lipid profiles,elevated FRS, and increased epicardial and liver fat [60–62]. Furthermore, VAT was independently associated with prevalent cardiovascular diseases in a study involving HIV-infected men [63]. More recently, Freitas et al. [64] reported a positive correlation beween VAT and carotid intima media thickness in HIV-infected patients with lipodystrophy. Tesamorelin has been shown to improve lipid profile by reducing triglyceride levels, without clinically meaningful changes in glucose parameters. While there was a statistically significant difference in HbA_1C_ elevation between the tesamorelin and placebo arms, no clinically significant changes in glycemic measures have been observed [30, 39]. Nonetheless, all patients treated with tesamorelin should be monitored periodically for changes in glucose metabolism. Administration of tesamorelin to HIV-infected patients with excess abdominal fat was recently shown to be associated with a significant decrease in liver fat. Interestingly, the reduction in liver fat was significantly associated with the reduction in VAT [65].

A recent exploratory analysis of the same Phase 3 data from over 800 patients found that the metabolic benefits of tesamorelin are limited to HIV-infected patients who respond to tesamorelin with a reduction in VAT [32]. Decreased VAT during tesamorelin therapy was associated with improvements in triglycerides, adiponectin levels, and preservation of glucose homeostasis. These benefits were not seen in individuals who do not respond to tesamorelin with a reduction in VAT. A recent study in HIV-negative patients showed a correlation between VAT reduction and the number of obesity-related risk factors with central adiposity [66]. However, this study involved all Asian patients, who accounted for only 5% of our study population. The current analyses differ from those published previously in that absolute change in VAT was examined to identify predictive factors. The rationale to select absolute change in VAT rather than percent change in VAT was based on the Phase 3 data showing that below a certain baseline VAT, patients had little, if any, VAT reduction with tesamorelin, despite the fact that all subjects had excessive accumulation of abdominal fat at baseline, as defined by pre-specified entry criteria [39]. This indicates that the actual measured VAT bears some value in determining how a patient will respond to tesamorelin.

The analyses presented here are pooled post-hoc analyses of two studies that were not originally designed to collect data specifically for calculations of the three composite risk scores (FRS, MetS-NCEP, and MetS-IDF). Therefore, it was necessary to derive some of the data elements needed for the composite risk scores and to conduct data review of some of the textual data collected to extract the needed data. Additional study limitations relate to the study population and ART regimens in this analysis. As the study population consisted largely of white male patients, our findings may not apply to other ethnicities or to women. Furthermore, most patients received older, less metabolically neutral ART regimens. Lastly, study eligibility criteria required all patients to have anthropometric parameters consistent with abdominal obesity for enrollment. Thus, it remains uncertain if only treated HIV patients with the excess VAT towards the higher end of the spectrum are those most likely to benefit from tesamorelin therapy.

In conclusion, patients with baseline MetS-NCEP, elevated triglycerides, or of white race are most likely to experience reductions in VAT after 6 months of therapy with tesamorelin. Furthermore, while overweight rather than obese individuals and those with VAT below 200 cm^2^ at baseline were most likely to achieve a reduction in VAT below the 140 cm^2^ threshold, it is currently unknown whether a clinical benefit can be achieved with any amount of VAT reduction.
